# Anti-Inflammatory Therapeutic Approaches to Prevent or Delay Post-Traumatic Osteoarthritis (PTOA) of the Knee Joint with a Focus on Sustained Delivery Approaches

**DOI:** 10.3390/ijms22158005

**Published:** 2021-07-27

**Authors:** Christine M. Khella, Judith M. Horvath, Rojiar Asgarian, Bernd Rolauffs, Melanie L. Hart

**Affiliations:** G.E.R.N. Center for Tissue Replacement, Regeneration & Neogenesis, Department of Orthopedics and Trauma Surgery, Faculty of Medicine, Medical Center—Albert-Ludwigs—University of Freiburg, 79085 Freiburg im Breisgau, Germany; christine.khella@outlook.com (C.M.K.); judith.m.horvath@gmail.com (J.M.H.); r0jiar.asgarian@gmail.com (R.A.); bernd.rolauffs@uniklinik-freiburg.de (B.R.)

**Keywords:** chondrocyte, articular cartilage, synovium, fat pad, osteoarthritis, post-traumatic osteoarthritis, immunomodulation, inflammation, degeneration, regeneration, anti-apoptotic, early PTOA, cartilage repair, clinical, knee trauma, knee joint, anti-inflammatory cytokines, cartilage, IL-1β, TNF-α, IL-6, triamcinolone acetonide, dexamethasone, IL-1 receptor antagonist, antibody, hyaluronic acid, IL-4, IL-10, IL-13, complement inhibitors, complement system, tranexamic acid, drug delivery, early disease, prevention

## Abstract

Inflammation plays a central role in the pathogenesis of knee PTOA after knee trauma. While a comprehensive therapy capable of preventing or delaying post-traumatic osteoarthritis (PTOA) progression after knee joint injury does not yet clinically exist, current literature suggests that certain aspects of early post-traumatic pathology of the knee joint may be prevented or delayed by anti-inflammatory therapeutic interventions. We discuss multifaceted therapeutic approaches that may be capable of effectively reducing the continuous cycle of inflammation and concomitant processes that lead to cartilage degradation as well as those that can simultaneously promote intrinsic repair processes. Within this context, we focus on early disease prevention, the optimal timeframe of treatment and possible long-lasting sustained delivery local modes of treatments that could prevent knee joint-associated PTOA symptoms. Specifically, we identify anti-inflammatory candidates that are not only anti-inflammatory but also anti-degenerative, anti-apoptotic and pro-regenerative.

## 1. Introduction

**Figure 1:**
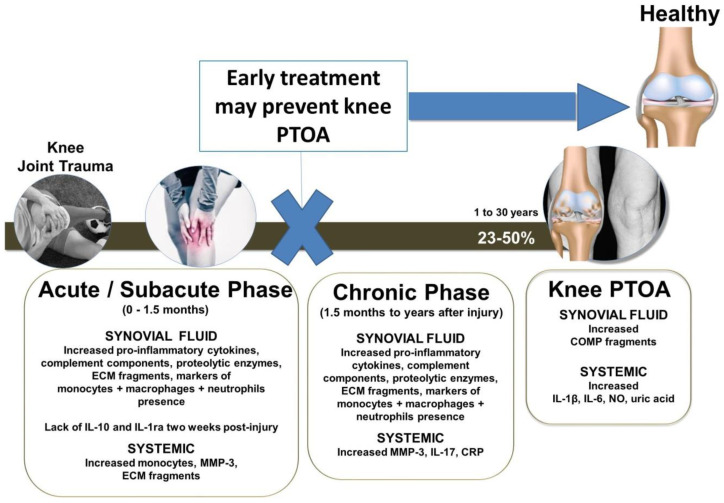
An early multifaceted anti-inflammatory therapeutic approach may reduce the continuous cycle of inflammation and articular cartilage degradation that often endures for months to many years following knee trauma. Whether resolution of chronic inflammation (indicated by the blue x) can be accomplished to prevent or halt the development of PTOA remains to be determined. This figure was adapted and summarized based on clinical data that demonstrated significant increases of these markers at the indicated time points after knee joint injury as discussed in detail in our systematic evidence-based review [3]. Abbreviations: cartilage oligomeric matrix protein (COMP), c reactive protein (CRP), extracellular matrix (ECM), matrix metalloproteinase 3 (MMP-3), nitric oxide (NO).

Osteoarthritis (OA) of the knee is one of the most common degenerative joint diseases and the most frequent cause of physical disability [1], affecting more than 650 million people worldwide [2]. The global prevalence of knee OA is 16% in individuals aged 15 and over and 23% in individuals aged 40 and over [2]. Post-traumatic osteoarthritis (PTOA) is a major cause of early knee OA [3] with 22–50% of people developing clinical symptoms of PTOA [4,5,6,7,8,9,10] within only a few years of an acute knee trauma [1,11,12,13,14,15] due to injuries such as intraarticular fracture, injury to the anterior cruciate ligament (ACL) alone or combined with medial collateral ligament or meniscus injuries [4,5,6,7,8,9,10]. This, as of yet, incurable, multifactorial chronic disease consists of multiple co-pathologies of the articular cartilage, synovial fluid, synovial membranes, and bone. Importantly, it is also connected with systemic manifestations of the disease, in which inflammation plays a key causal role in inducing knee PTOA disease progression [3]. This systemic response begins within 1 week of knee trauma, leading to higher than normal levels of inflammatory and injurious biomarkers present in the synovial fluid, blood and urine, and can persist for many years after knee injury (Figure 1). A long-term continuous low-grade inflammation concomitant with the local presence of various immune system mediators including inflammatory cytokines, neutrophil and macrophage-associated factors, complement system activation products, cartilage-degrading proteolytic enzymes as well as ensuing mediators of articular cartilage degradation can endure for months to many years following knee trauma, indicative of early, progressive, and conceivably irreversible, damage to the cartilage tissue [3].

Recently, the aim of treating PTOA of the knee has shifted from late-stage treatment to preventing or delaying early disease progression. Recommendations were made for delaying or preventing PTOA in exercise- and sports-related injuries that favor promoting early exercise therapy to increase muscle strength and neuromuscular control after joint trauma [16]. This approach can minimize the risks factors of PTOA such as weak musculature, elevated adiposity, and physical inactivity, and may even stimulate better regeneration of the tissues, while simultaneously downregulating pro-inflammatory and proteolytic pathways [17]. However, non-compliance with physiotherapy along with poor adherence to exercise is common [18]. Therefore other strategies, outside of the physiotherapy-based standard of care, are needed to address the persistent inflammation that promotes the degradation of articular cartilage and other tissues and PTOA progression.

Considering the large role that inflammation plays in the pathogenesis of knee PTOA after knee trauma [3], there may be a window of opportunity, in which anti-inflammatory therapeutics are applied early following knee joint injury to prevent inflammation and the downstream effects that lead to PTOA of the knee (Figure 1). This review focuses on identifying the optimal timeframe of a potential application of local intra-articular (i.a.) anti-inflammatory treatments that can (i) control inflammation, (ii) prevent articular cartilage damage, (iii) reduce cell death, but also (iv) promote the regeneration of the articular cartilage of the knee joint. Sustained delivery of anti-inflammatory drugs over a longer period of time may overcome problems such as temporary effects due to short half-lives and rapid efflux from the knee joint into the systemic circulation that are associated with both current clinically approved disease-modifying OA drugs (DMOADs), including dexamethasone and triamcinolone acetonide [19,20], and not yet clinically approved drug candidates [21,22,23,24]. Therefore, this review also discusses sustained delivery approaches that were tested in the knee joint or in articular cartilage injury/inflammatory models. This would not only increase the therapeutic joint residence time but would also abolish the need for successive i.a. injections that are needed for beneficial effects as prolonged exposure or successive i.a. injections may cause damage to the articular cartilage. In addition to reviewing the available DMOADs, which were proposed to provide anti-inflammatory effects, we also focus on studies involving hyaluronic acid, specific inhibitors of TNF-α, IL-1β, IL-6 and the complement system, the anti-inflammatory cytokines IL-4, IL-10, and IL-13, and the anti-fibrinolytic tranexamic acid. While the knee joint is of particular interest to this review, it is evident that only a limited number of past or present clinical trials have tested these agents clinically to target and clinically prevent or impede PTOA knee joint progression (Table 1), and, therefore, trials focusing on other joints have also been included but labeled accordingly. With an emphasis on early disease prevention, state-of-the-art studies in animals involving the knee joint, ex vivo articular cartilage and in vitro models are also included.

## 2. Methodology

Eligible articles were identified using PubMed, Medline, Cochrane Library, Google Scholar, Web of Science databases, and by hand-searching. The following inclusion criteria were used: (1) peer-reviewed studies that were published in the English language, (2) anti-inflammatory therapeutic intervention studies that claimed to investigate or prevent inflammation related to the knee joint or the cells or tissues thereof, and, unless otherwise stated, (3) in vitro or ex vivo explant models that used cells or tissue that was obtained or isolated exclusively from the knee joint, and (4) in vivo PTOA models of the knee joint that applied insult/injury and/or inflammatory cytokines (chemically-induced rheumatoid arthritis (RA) models of injury were excluded). There were no exclusion criteria based on the date of publication.

## 3. A Brief Description of the Cells and Tissues That Participate in the Pathogenesis and Progression of PTOA within the Knee Joint

**Figure 2:**
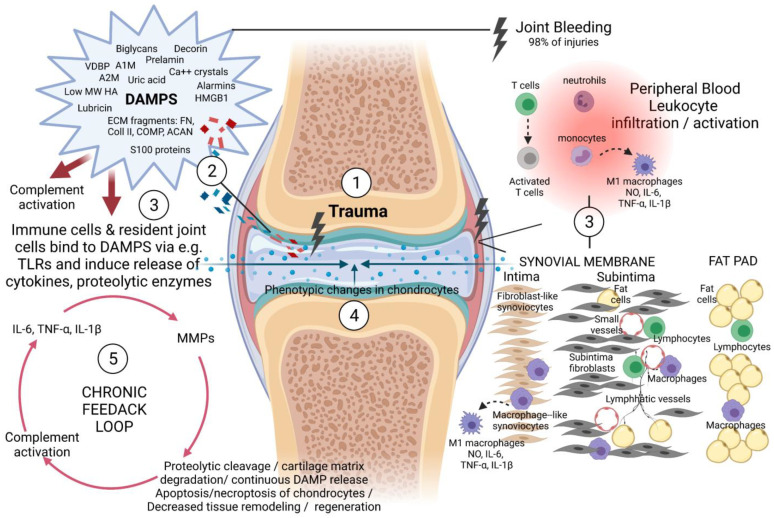
The major cells, tissues and factors involved in the trauma-associated response following knee joint injury and chronic inflammation of the knee joint. In OA [42,43] and (1) trauma-associated conditions (e.g., mechanical injury, joint bleeding) [44,45], (2) damage-associated molecular patterns (DAMPS) are released from injured cells and tissues, which (3) mainly drives the inflammatory response in the cells of the synovial tissue [26,34,35,36] but also chondrocytes [3], IFP cells [38,39] as well as in the innate immune cells (peripheral blood leukocytes including T cells [32,46,47,48] and monocytes/macrophages [3]). DAMPs bind to cells through pattern recognition receptors (PRRs) including toll-like receptors (TLRs), NOD-like receptors (NLRs), and receptors for advanced glycosylation end products (RAGEs). This leads to activation of signaling pathways that cause the production of various factors including pro-inflammatory cytokines (IL-6, TNF-α, IL-1β), chemokines that, in turn, further recruit leukocytes to the site of injury, catabolic factors (MMP-1, MMP-3, MMP-9, MMP-10, and the intracellular catabolic procathepsin B that can be activated by GAGs [49]) and activation of the complement cascade [43,45]; factors described as essential in PTOA knee pathogenesis [3]. Hence, the generation of DAMPs that are produced by trauma or enzymatic degradation that turn immunologically quiescent ECM components into fragments that activate PRR signaling pathways are referred to as extracellular DAMPs. Those associated with OA/PTOA (reviewed in [42,43]) include fragments of aggrecan (ACAN); collagen type II (Coll II); cartilage oligomeric matrix protein (COMP); low mw hyaluronic acid (HA); biglycan and decorin that belong to the small leucine-rich repeat proteoglycan (SLRP) family; and lubricin. DAMPs can also be intracellular molecules that are released during cell necrosis including prelamin located in the inner nuclear membrane; S100 proteins: S100A8 and S100A9 released by monocytes, activated macrophage and neutrophils; the high mobility group box protein 1 (HMGB1) nuclear protein released by necrotic cells or secreted by macrophages in response to inflammatory cytokines TNF-α and IL-1β; uric acid (the metabolic breakdown of purine nucleotides), and calcium (Ca^+^)-containing crystals [calcium pyrophosphate dehydrate (CPPD) and basic calcium phosphate (BCP)]), and even plasma proteins (α1-microglobulin (A1M); α2-macroglobulin (A2M); and Gc-globulin also known as vitamin D-binding protein, VDPB) (reviewed in [42,43]). (4) These pathogenic effects cause phenotypic changes of chondrocytes, cell death and proteolytic enzyme production, which may further drive cartilage degradation [26,27,28,29] and, hence, DAMP production. The DAMP/PRR response is a normal response that serves as a defense strategy for maintaining and restoring homeostasis, but it can also become (5) dysregulated if DAMPs continue to be produced, resulting in inflammatory and tissue repair processes that may become pathogenic [44]. If the inflammatory process is not resolved, a chronic feedback loop that involves cross-talk and feedback among the cells within these tissues continues and may lead to PTOA progression due to continuous proteolytic cleavage of the ECM, cell death and continued activation of the innate and adaptive immune response [3].

Various tissues of the knee joint work together to orchestrate inflammation/resolution of inflammation and tissue repair/tissue degeneration including the cells within the synovial membrane (synovium), infrapatellar fat pad (IFP) as well as immune cells such as macrophages, lymphocytes, and other immune cells that invade the injured site or enter through the vasculature of the synovial tissue (Figure 2) [26,27,28,29,30,31,32]. The inner synovial membrane of the joint capsule encapsulating the entire joint cavity consists of two layers, the (i) synovial lining (intima) made up of two to three continuous layers of cells (20–40 mm thick) consisting primarily of CD14^−^ fibroblast-like synoviocytes (FLS, or Type B synoviocytes) that produce the synovial fluid as well as some resident CD14^+^ macrophage-like synoviocytes (MLS, or Type A synoviocytes) and the (ii) synovial sublining (subintima), which is approximately 5 mm thick in healthy tissue and contains small blood and lymphatic vessels [33] as well as synovial fibroblasts, fat cells, and a few macrophages and lymphocytes [31,34,35,36]. The IFP is interposed between the joint capsule and the synovial membrane [37] and is composed primarily of adipocytes but also MSCs and various immune cells such as macrophages and T cells [38,39]. The synovial membrane and the IFP are proposed to function as an anatomo-functional unit through the mutual interplay and the release of pro-inflammatory mediators, the induction of tissue modifications such as fibrosis [37], and partially responsible for the post-injurious pro-inflammatory response and development of PTOA, including in young adults [40,41].

## 4. Anti-Inflammatory Therapeutic Interventions to Prevent or Treat PTOA of the Knee Joint

### 4.1. Dexamethasone

A therapeutic agent that has received much attention in the treatment of OA, as well as PTOA, is dexamethasone. Dexamethasone is a wide-spectrum steroid glucocorticoid that has been shown to have anti-inflammatory effects and can improve proteoglycan synthesis and decrease GAG loss in injured cartilage in bovine and human explants in vitro [50,51,52,53]. Overall, dexamethasone was shown to have various degrees of protection in rodent (rat, mouse, and rabbit) inflammatory (i.e., using i.a. injection of TNF-α) or PTOA injury models by inhibiting matrix loss of articular cartilage and synovial and fat pad inflammation and by decreasing the histological grade of OA/PTOA [54,55,56,57,58], suggesting that dexamethasone could have a disease-modifying effect. For example, in an osteochondral drill injury rabbit PTOA model a single i.a. injection of low dose dexamethasone given 48 h after surgery significantly decreased IL-1β, IL-6 and IL-8 as well as matrix metalloproteinases (MMP-1, -3 and -13) mRNA levels 2 months post-surgery and resulted in a better histological grade [56]. Using the same PTOA model, repeated i.a. injections of dexamethasone given every three days for three weeks after drill-induced injury significantly suppressed the expression of IL-1β and COL1 and showed a trend in decreasing MMP-3 in the synovium, while in the fat pad dexamethasone showed a trend toward reducing IL-1β expression and significantly decreased the expression of both MMP-13 and basic fibroblast growth factor (bFGF) [55], which may contribute to OA progression [59]. Combined oral dexamethasone and indomethacin, a nonsteroidal anti-inflammatory drug (NSAID), was also capable of decreasing synovial inflammation in a medial collateral ligament transection (MCLT) rat PTOA model [54]. Collectively, this suggests that dexamethasone could act on both synovial and IFP cells.

In vitro and ex vivo human and bovine cartilage explant inflammatory (using, e.g., IL-1α or TNF-α) or injurious compression models have shown similar benefits of dexamethasone, including inhibition of pro-inflammatory cytokine and MMP synthesis, as well as prevention of nitric oxide (NO) production, glycosaminoglycans (GAG) loss while increasing aggrecan and COL2 expression (dexamethasone in combination with insulin growth factor-1 (IGF-1) in bovine tissues) and synthesis of GAGs [51,52,53,60]. An in vitro articular chondrocyte study showed that dexamethasone inhibited NO, IL-1 and IL-6 production when administered prior to IL-17 stimulation [61]. Dexamethasone was also capable of decreasing the gene expression of IL-1β, TNF-α, IL-6 as well as MMP-9 and iNOS from interferon-gamma (IFN-γ)/lipopolysaccharide (LPS)-polarized M1 macrophages [62].

A few studies have shown that dexamethasone, when covalently linked to the small, highly cationic molecule avidin with a molecular weight (*M*_W_) of 66 kDa and a diameter of 7 nm, allowing diffusion and penetration into the articular cartilage [63,64,65], resulted in a prolonged decrease in joint inflammation up to 50% 3 weeks after anterior cruciate ligament transection (ACLT) injury in rabbits, compared to a single i.a. bolus of dexamethasone alone [53,66]. Avidin-dexamethasone treatment also led to decreased gene expression of IL-1β, MMP-1, and ADAMTS-5 and restored ACAN to normal expression levels but was unable to prevent MMP-3 and -13 expression and GAG loss [66]. This group further optimized the release of dexamethasone by attaching dexamethasone to avidin-biotinylated polyethylene glycol (PEG) using hydrolyzable ester linkers that allowed both a fast burst and a slower and sustained release of dexamethasone within healthy as well as partially degraded bovine articular cartilage tissue for up 2 weeks that reduced IL-1α-induced cell death and loss of GAGs [53,65].

A clinical phase II dose-finding study of TLC599 (NCT03005873), a liposomal (lipid-based) formulation that encapsulates dexamethasone and provides sustained release of dexamethasone, resulted in a significant decrease in pain in patients with clinical knee OA receiving a single i.a. TCL599 injection compared to the control group. The pain was reduced throughout the 24 week trial period and no treatment-related serious side effects were recorded [67]. While this study did focus on the knee joint, trial subjects were exclusively patients over the age of 50 with at least 6 months of diagnosed OA, and the primary aim was pain reduction rather than disease treatment. Nevertheless, this study indicates both a high safety of sustained treatment with dexamethasone, suggesting a possible application to knee PTOA patients. A pilot clinical trial using dexamethasone with the intention of preventing the progression towards PTOA after injury to the wrist was launched in 2014 and was completed, but as of now, results are unavailable (NCT02318433). In this trial, the aim was to test for a reduction in the onset of PTOA in patients with a distal radius fracture who were given a single i.a. injection of dexamethasone after injury. This is particularly relevant because it focuses on the prevention of PTOA progression, thereby aiming at modulating the disease while adhering to the window of opportunity previously discussed.

**Figure 3:**
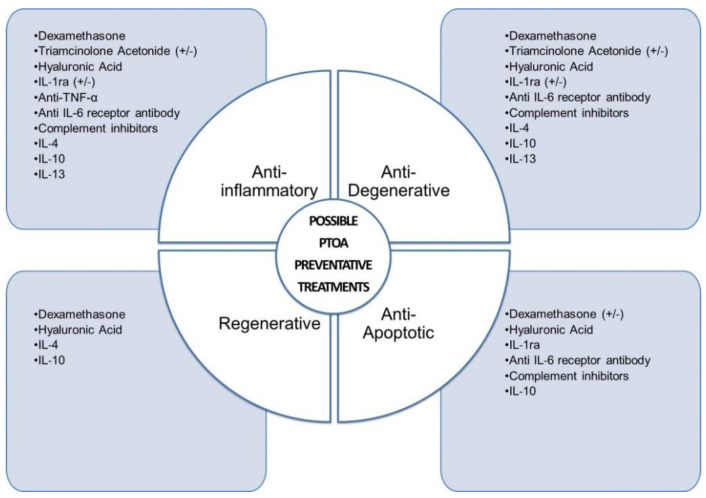
Possible treatment interventions that could prevent or delay PTOA-driving processes in the knee joint by suppressing inflammation, degeneration and apoptosis, while activating regenerative processes. All of the therapeutic candidates shown here exert anti-inflammatory effects by decreasing the injury/inflammation-related joint swelling, synovial inflammation and/or levels of pro-inflammatory mediators. Although some agents have anti-degenerative activity, and inhibit cartilage degradation following injury by, e.g., decreasing the expression of MMPs as well as the loss of GAG, anti-TNF-α showed adverse effects on cartilage and bone and increased inflammation. Few of these agents have regenerative effects that are capable of enhancing the chondrogenic properties of injured cartilage. Similarly, only a few agents counteract the apoptosis of chondrocytes. Only hyaluronic acid and IL-10 possess all four main therapeutic potentials against PTOA development. The (+/−) sign indicates conflicting reports, based on knee joint-related clinical, articular cartilage ex vivo and/or in vitro studies.

Overall, the data discussed in this section suggest that dexamethasone has anti-inflammatory, anti-degenerative, and pro-regenerative effects in the knee joint (Figure 3). However, in regard to averting cell death, the effects of dexamethasone were contradictory with some studies showing that it prevents cell death and other studies showing that it has cytotoxic and catabolic side effects, resulting in a significant increase in chondrocyte apoptosis and a significant decrease in chondrocyte proliferation [57,68,69]. While this was shown to occur to a greater degree with higher doses and repeated injections, this could theoretically exacerbate damage to the articular cartilage and accelerate the progression towards clinical PTOA. Therefore, the appropriate dosage, duration and timing of treatment need to be investigated in the specific types of knee joint injuries for which dexamethasone usage is intended to ensure that the benefits outweigh the potential harm of dexamethasone.

### 4.2. Triamcinolone Acetonide (TCA)

TCA (Kenalog-40^®^ or Kenacort-A 40^®^) is another clinically approved glucocorticosteroid used for i.a. treatment of OA-associated knee pain [70]. Evidence from clinical trials has shown mixed effects, with some studies revealing that long-term consecutive TCA treatment (e.g., given every 3 months for up to 2 years) causes articular cartilage damage and disparities in controlling knee pain [71]. TCA (i.a.) has also been linked to the presence of several adverse systemic effects including elevated blood glucose levels [72]. Nevertheless, a few in vivo studies showed that TCA reduced cartilage degradation when given shortly after a knee injury. Lattermann et al. investigated the use of i.a. TCA (Kenalog-40^®^) administered to patients either 4 days or 2 weeks or at both time points after ACL injury and showed that, after 5 weeks, the C-terminal cross-linked telopeptide of type II collagen (CTX-II), which is associated with type II collagen degradation, was significantly decreased in the synovial fluid in both of the groups that were treated early (i.e., 4 days) after injury. Synovial fluid cartilage oligomeric matrix protein (COMP) levels were also significantly decreased in the group treated with TCA 2 weeks after injury. However, 5 weeks after treatment patient-reported outcomes did not differ between any of the patients receiving TCA vs. the placebo group and there was a minimal but non-significant reduction of other synovial fluid chondrodegenerative (i.e., N-terminal telopeptides of type I collagen (NTX-I), and MMP-1, -3, -9) or inflammatory (TNF-stimulated gene 6 protein (TSG-6), IL-1α, IL-1β, IL-1ra) markers [73]. Similarly, Sieker et al. used a porcine PTOA ACLT injury model to examine the effect of a single i.a TCA injection immediately after injury. This group showed that compared to the ACLT group without treatment, 14 days of TCA treatment significantly decreased the concentrations of C1, C2 collagen fragments in the synovial fluid and, in the synovial membrane lining, the number of mononuclear leukocytes, as well as the expression of the CX3CR1 chemokine receptor, which is found on synovial macrophages, dendritic cells, T cells and synovial fibroblasts [74], was reduced. Surprisingly, compared to the ACLT group, the expression of neutrophil/macrophage-associated MMP-8 was significantly increased in the TCA-treated group. Similar to the aforementioned study, TCA did not reduce MMP-1 and -2 [75]. Another group showed that continuous bolus TCA treatment of compressively injured articular cartilage explants obtained from young (1–2 week old) healthy bovine knee joints significantly decreased sGAG loss in the presence of inflammatory mediators (TNF-α, IL-6, and soluble IL-6 receptor) [76]. These data collectively suggest that TCA has cartilage protective effects when given after knee trauma but the results on regulating MMPs and inflammation are ambiguous.

Due to the short-termed joint residence time and fast clearance of TCA from the joint [19,20], polyester amide (PEA) [77,78] and poly lactic-co-glycolic acid (PLGA) [79] microsphere-mediated sustained-release TCA systems were developed. PEA and PLGA microspheres resulted in a burst release of TCA followed by a steady-state release of TCA in PBS for 40–60 days [77,78]. In a rat OA model induced by injecting the patellar ligament with type II collagenase, i.a. retention of PEA microspheres (given 1 week after injury) was observed for up to 8 weeks after treatment with a gradual decline of signal over time. Two of six rats still had PEA microspheres present 8 weeks post-injury. While TCA did not improve the cartilage integrity (Mankin scores) with either bolus or PEA microsphere-mediated delivery of TCA, synovial inflammation (Krenn scores; synovial lining thickness and inflammatory infiltrate) was significantly reduced in OA joints receiving the microsphere sustained TCA release system but not by bolus TCA. TCA administered with microspheres also significantly decreased TNF-α induced prostaglandin E₂ (PGE2) in human chondrocytes [77]. In a repeated synovitis rat acute arthritis model induced by a single i.a. injection of streptococcal peptidoglycan-polysaccharide (PGPS) followed by i.v. PGPS injections each week for 4 weeks, TCA (Kenacort-A 40^®^) loaded PEA microspheres decreased synovitis (Krenn scores) more than TCA loaded PLGA (Resomer^®^) microspheres or bolus injection of TCA. However, none of the TCA treatment strategies decreased cartilage histology (Mankin) scores. Interestingly, lameness was more frequent in rats treated with TCA-loaded PLGA microspheres compared to PEA microspheres [78]. This may be due to the different material properties or degradation and erosion techniques, e.g., PLGA degrades by bulk erosion, while PEA degradation is mediated by serine protease enzymatic activity [80,81]. PEA-mediated TCA delivery showed a significant difference compared to bolus administration for several pain parameters, whereas almost none differed when the PLGA microspheres were compared to a bolus injection of TCA, together indirectly suggesting that PEA-mediated delivery of TCA was more effective than PLGA (Resomer^®^) delivery of TCA [78]. However, in the same acute repeated synovitis rat model, sustained release of TCA following a single i.a. injection of TCA-releasing PLGA microspheres (FX006, Zilretta^®^) significantly improved histological (inflammation, pannus, cartilage damage, and bone resorption) scores compared to vehicle or TCA (Kenalog-40^®^) bolus treatment alone [79]. This suggests that different microsphere formulations may have different effects depending on the system. It also shows that, while these models were not PTOA models per se, sustained microsphere-mediated release of TCA decreased inflammation of the knee joint but had no effect on preventing cartilage damage under OA-like [77] or acute cyclic bacterial-mediated synovitis-like conditions [78,79].

Clinically, the PLGA microsphere injectable FX006 (Zilretta^®^) suspension for extended TCA release was FDA approved in 2017 for i.a. injection management of OA-associated knee pain [70]. This PLGA-release system was shown to increase the residence time of TCA in the human knee joint for up to 12 weeks [82]. In another study, repeated i.a. injection of TCA (FX006) reduced pain by half for 74% of patients [83]. Concerning knee PTOA prevention and preventing the advancement of knee joint disease, a current Phase 2a clinical trial (NCT04331002) is investigating whether these PLGA TCA-releasing microspheres improve patient-reported outcomes, decrease progressive bone shape changes, degradation of articular cartilage (CTX-II release) and persistent inflammation such as high levels of synovial fluid IL-6 in patients with ACL reconstruction with meniscal involvement. Another clinical trial is focusing on whether this sustained TCA release system is capable of providing pain relief and enhancing function in patients already diagnosed with mild to moderate knee PTOA (Table 1).

### 4.3. Hyaluronic Acid (HA)

Normal synovial fluid contains 1 to 4 mg/mL of HA (also known as hyaluronan), a polymer of repeating disaccharide units of N-acetylglucosamine (GlcNAc) and glucuronic acid that is produced and extruded into the synovial fluid by synoviocytes, fibroblasts, and articular chondrocytes [84,85,86]. At the articular surface, HA binds and retains aggrecan to the plasma membrane of chondrocytes, forming a massive network of aggregates of fixed negative charge allowing the extracellular matrix (ECM) to retain water and retard the diffusion of large molecules [85,86,87,88,89] while helping to maintain normal cell–cell spacing distances between superficial zone chondrocytes in cartilage tissue [90]. Therefore, it was suggested that HA not only maintains the synovial fluid viscosity but also provides boundary lubrication, shock absorption, elasticity and hydration as well as joint homeostasis. Native HA in the adult knee has a molecular weight (*M*_W_) of 4000 to 10,000 kDa [86,91]. During inflammation and/or injury excess HA fragmentation and accumulation of fragments occurs, which markedly decreases the concentration of HA in the synovial fluid, due to its higher rate of clearance compared to non-fragmented HA. Although a mechanism has not been described in the knee joint, it was shown that macrophages are capable of internalizing HA in a CD44-dependent manner, which is then delivered to the lysosomes and subsequently degraded [92]. This response was shown to occur in response to mechanical injury (compressive loading to the knee joint or traumatic injury) and the presence of inflammatory cytokines such as IL-1β and TNF-α. For example, chondrocytes directly within a mechanically-injured area do not express CD44 [93,94], likely due to endocytosis-mediated internalization of CD44, which correlates with the local presence of MMPs and aggrecanases (i.e., a disintegrin and with thrombospondin motifs-4 and -5, ADAMTS-4 and -5) within the pericellular matrix (PCM) [90]. 

During OA disease progression, depolymerization of endogenous HA occurs and causes the higher molecular mass forms of HA normally present in the joint to be converted into lower *M*_W_ forms, which also contributes to inflammation by binding to several cell surface receptor complexes such as CD44, TLR2, and TLR4 on, e.g., chondrocytes and macrophages, causing downstream signaling and cytokine release [85,86,87,88,95]. These modifications in both the structure and concentration of HA, in turn, reduces HA’s ability to lubricate the joint surface and distribute weight-bearing stresses due to changes in the boundary lubricating layer complexes consisting of HA, lubricin molecules and phosphatidylcholines lipids [96,97]. These altered complexes are more easily removed (e.g., due to shear stresses) when the HA mediating their attachment is of lower *M*_W_, which could cause increased friction and thus enhance degradation of healthy cartilage. For these reasons, injectable HA formulations were assessed for clinical use in OA (e.g., Hyalgan, HYADD4-G). However, it is important to note that a wide array of HA preparations are commercially available, which are derived from different sources, have different molecular weights and different degrees of cross-linking making joint residence times, rheological properties and cross-comparisons difficult [20,84].

Various clinical studies are currently focusing on testing the safety and efficacy of i.a. HA injections into the knee joint over a long timeframe. However, as most clinical trials of HA have so far focused on patients older than 40 years with knee OA (e.g., [98], NCT02280538), it is difficult to determine their possible benefit on early PTOA prevention in humans. Nevertheless, a study on knee OA by Listrat et al. did show a reduction in the progression of the disease by visual assessment of chondropathy during arthroscopy. Three i.a. Hyalgan injections, which are composed of highly purified linear HA with an average-molecular-weight of 500 to 730 kDa and a very low elastic modulus, were given over the course of two weeks every three months over a period of one year. X-ray analysis did not show significant differences in joint space narrowing between the treatment and control group, but joint deterioration appeared to occur to a lesser extent in the treatment group [99]. Although this study is limited by the small number of patients and the lack of quantitative and sensitive outcome measurements such as MRI, it nonetheless might present some evidence for the disease-modifying capabilities of HA.

Another study on knee OA compared the effect of i.a. injection of Hyalgan once a week for five consecutive weeks to i.a. injection of methylprednisolone acetate (Depo-Medrol), a synthetic glucocorticoid corticosteroid that was given once a week for 3 consecutive weeks. This study is particularly interesting because it separated patients into knee OA and PTOA groups. Visual assessment via arthroscopy and electron microscopy of synovial membrane biopsies prior to treatment showed different synovial membrane structural features in OA vs. PTOA patients compared to controls (patients who had undergone arthroscopy for pain but without any arthroscopic sign of OA or RA). In untreated patients, the synovial membrane of primary OA patients exhibited a modest inflammation, with macrophages present in approximately 80% of the patients and a 2.5-fold increase in mast cells and lymphocytes compared to controls. PTOA patients showed more intense inflammation and associated reparative fibrosis, with a higher number of macrophages and fibroblasts, as well as large, thick collagen bundles and a high number of ectasic vessels in both the sublining and subsynovial regions of the synovial membrane. Reassessment of patients six months after the start of the trial demonstrated that, in both OA and PTOA patients, HA was more efficient than Depo-Medrol in reducing the number and clustering of B synoviocytes (synovial fibroblasts) and increasing pinocytic vesicles that line the plasma and cytoplasmic membranes of these cells, indicative of healthy cells in the synovial lining. A significant decrease in inflammation with both treatments in characteristics such as cellularity, often associated with the increased presence of inflammatory cells, was also observed for both OA and PTOA patients. However, the effect was more evident in the OA group vs. the PTOA group [100].

In vitro [62,101] and in vivo studies in animals have highlighted the anti-inflammatory and chondroprotective effects of HA derivatives and their impact on synoviocytes, chondrocytes and macrophages. One study using different concentrations of the amine HA derivative (500 to 730 kDa) hydrogel called HYADD 4-G combined with platelet-rich plasma obtained from mouse blood, which was applied i.a. in a non-invasive single axial tibial mechanical loading mouse model, did not show protective effects on proteoglycan, cartilage oligomeric matrix protein (COMP) and aggrecan loss, or in preventing chondrocyte apoptosis or synovitis at 5 and 56 days post-injury [102]. In contrast, multiple other animal studies have demonstrated the effectiveness of i.a. HA injections in reducing cartilage degeneration and enhancing chondroprotection in knee PTOA animal models [103,104,105]. These and many other studies have shown that higher *M*_W_ HAs were generally more effective in reducing synovial inflammation and restoring the visco-induction of synovial fluid than HAs with lower *M*_W_s [86,91]. In IL-1β-stimulated human synoviocytes isolated from patients with a tibial plateau fracture, both high and low *M*_W_ HA decreased IL-1β and TNF-α and induced production of the anti-inflammatory cytokine IL-10 as well as tissue inhibitor of metalloproteinases (TIMP-1). Interestingly, high *M*_W_ HA was more effective in reducing IL-1β and TNF-α, while low *M*_W_ HA increased MMP-1 and-2 and decreased MMP-3 [101]. In a transwell co-culture model containing early OA articular chondrocytes and M1 macrophages from a human monocyte-derived THP-1 cell line used to resemble the permeable synovial joint, high *M*_W_ HA significantly reduced secretion of IL-1β and TNF-α, but not IL-6 from OA chondrocytes. The high-*M*_W_ HA also significantly increased the mRNA expression of COL2A1 and reduced the expression of COL1A1 and MMP-3 in OA chondrocytes [62]. Another study showed that in rat chondrocytes different concentrations of HA (with a low to medium *M*_W_ of 500–730 kDa) decreased and prevented IL-1β induced apoptosis in a dose-dependent manner [106]. The beneficial effects of HA (summarized in Figure 1) warrant further clinical investigations with a focus on PTOA in a time frame closer to joint injury.

### 4.4. Inhibitors of TNF-α and Interleukin-1 Receptor Antagonist (IL-1ra)

We recently used the Bradford Hill Framework [107] for evaluating epidemiologic evidence of a relationship between the presence of inflammatory markers after knee trauma and PTOA disease progression [3]. By evaluating both clinically orientated studies and knowledge derived from basic science studies, we identified the inflammatory cytokines TNF-α and IL-6 as causal factors and IL-1β and IL-17 as credible factors in inducing knee PTOA [3], making them attractive targets for PTOA prevention. While TNF-α is clearly an important pro-inflammatory cytokine involved in the pathogenesis of PTOA of the knee and is present within 24 h and up to 5 years after knee trauma [3], there have not been any clinical studies that have solely focused on early TNF-α inhibition in the context of knee joint inflammation. This may be due to evidence showing that systemic (oral) anti-TNF-α therapy in RA patients was shown to increase the risk of infections and malignancies by causing an overall immunosuppressive state [108] and that sustained i.a. delivery of a TNF-α inhibitor in a mouse tibial plateau fracture injury model negatively affected cartilage and bone healing [109,110].

However, there were several pilot studies on the use of adalimumab (Humira^®^) and infliximab (Remicade^®^) in patients diagnosed with knee OA [111,112,113,114]. Those two substances are the two most commonly used chimeric monoclonal antibodies against TNF-α, which are used in clinical practice worldwide to treat inflammatory diseases such as RA, ankylosing spondylitis, psoriatic arthritis and Crohn disease [115,116]. As in many other studies, joint trauma was an exclusion criterion and, therefore, the effect after knee trauma was not addressed. The focus of these studies was mostly on pain reduction in OA rather than prevention or reversal of disease effects. Patients with advanced radiological (Kellgren–Lawrence Score (KL) grade 2 or grade 3) evidence of knee OA receiving six biweekly subcutaneous (s.c.) injections of adalimumab over 12 weeks showed a significant improvement in WOMAC scores, stiffness, function, and joint swelling at 12 weeks. However, 22 weeks after treatment discontinuation, beneficial effects were only still evident in 50% of patients (NCT00686439) [111], likely due to the advanced stage of the disease. Adalimumab was also used to treat severe knee OA and after 4 weeks, i.a. injection led to better outcomes in terms of pain and function compared to HA treatment. However, a placebo group was not included in this study [112]. A clinical phase I and II trial (NCT00819572) investigated the safety and efficacy of i.a. injection of DLX105 (ESBA105^®^), a single-chain antibody fragment against TNF-α, to reduce pain in the knee joints in patients with minimal to moderate OA (KL grades 2–3), according to radiographs, and showed that the total WOMAC score significantly improved at day 56 [113]. The results of i.a. DLX105 treatment were also evaluated in 2010 in a clinical trial on patients with OA of the knee (NCT00819572) but the results are not yet published. In vivo, i.a. DLX105 inhibited TNF-α induced synovial inflammation in rat knee joints with an efficacy similar to infliximab and resulted in a significant (90%) inhibition of knee joint swelling, inflammatory infiltrates as well as proteoglycan loss 48 h after treatment [21]. DLX105 (26 kDa) was also able to penetrate into bovine healthy articular cartilage explants as well as into the synovial tissue, cartilage and surrounding tissues after in vivo administration, while infliximab (an anti- TNF-α IgG of ~150 kDa) was unable to penetrate into the articular cartilage and remained on the cartilage surface of cartilage explants. However, the half-life of DLX105 is only 4–24 h, suggesting short-term effects [21]. Another trial (NCT01144143) focused on the ability of infliximab to prevent further cartilage degeneration in patients already diagnosed with early OA in the knee joint in which a single i.a. injection was given and the patients followed for a total of 10 weeks. The study was completed in 2018 and the results have not been published. Infliximab was also tested in patients having undifferentiated arthritis, RA, psoriatic arthritis, spondylarthropathy, or juvenile chronic arthritis with persistent inflammation despite one or more steroid injections within a year. While this study excluded patients with knee OA, gout or infection, two consecutive i.a. treatments over the course of 3 months was not effective at reducing signs or symptoms of inflammation or delaying the need for additional treatments [114], which may be due to the lack of penetration into the tissue [21].

There has also been a great deal of focus on using interleukin-1 receptor antagonist (IL-1ra), the natural inhibitor of IL-1β that competes with IL-1β for the occupation of surface IL-1 receptors and thereby prevents downstream signaling of IL-1β [117], to prevent PTOA progression. After acute knee trauma, the concentrations of IL-1ra generally increase in the synovial fluid within the first two weeks after injury, but then IL-1ra becomes undetectable, while, in contrast, IL-1β remains significantly elevated up to 1.5 months after knee trauma and rises again once clinical PTOA of the knee is diagnosed [3], suggesting that IL-1ra supplementation may be beneficial in preventing knee PTOA. Moreover, an allelic polymorphism in intron 2 of the IL1-ra gene (IL1RN*2) predisposes to the development and/or severity of a variety of human inflammatory diseases and was found in 21.4% of the population [118], with some studies showing an associated with knee OA [119] and other studies showing a lack of association [120], depending on the patient cohort.

A phase II clinical study showed that i.a. injection of IL-1ra significantly improved knee pain 4 days after treatment in patients having knee OA. However, the effect was lost after 1 month and the study concluded that a single i.a. injection of IL-1ra had no effect on decreasing knee pain, function, stiffness, or cartilage turnover [121]. Another clinical trial focused specifically on preventing sustained inflammation following knee trauma, as IL-1ra was administered via i.a. injection at a mean of two weeks post-injury (ACL tear) and patients were assessed at a mean of 35 days post-injury [25]. While no adverse reactions were reported and a significant decrease in synovial fluid IL-1α and systemic serum HA was observed, there was no effect of IL-1ra in decreasing IL-1β concentrations in the synovial fluid. A current phase 2 clinical trial is investigating whether i.a. injection of IL-1ra (Anakinra^®^, Kineret^®^) will decrease the risk of developing early PTOA in individuals (age 14–40 years) with an acute ACL tear and painful effusions by treating within 28 days of injury (NCT02930122). The outcome measures of this study are CTX-II levels from injury to time of surgery as well as Knee Injury and Osteoarthritis Outcome Score (KOOS) scores and quantitative T1rho magnetic resonance imaging (MRI) 1 year after injury. Although promising, a major shortcoming of these studies is the short timeframe and the use of a single treatment. A current phase 1 trial (NCT04119687) in patients having KL grades 2–4 knee OA addresses this point by investigating the safety and tolerability of an IL-1ra gene therapy approach (FX201 from Flexion), which is a helper-dependent adenovirus (HDAd) gene transfer vector that codes for IL-1ra in the presence of inflammation via a nuclear factor-kappa B (NF-kB)-responsive promoter.

A number of studies have investigated the use of IL-1ra (Anakinra^®^, Kineret^®^) and/or soluble TNF receptor II (sTNFRII, Enbrel^®^ or Etanercept^®^) that sequesters TNF by competitively binding TNF-α and TNF-β and thereby prevents their binding to the TNF receptor [122], in various animal models. These studies showed, depending on the mode of treatment, more beneficial effects with IL-1ra treatment [110,123,124,125], but adverse effects using sTNFRII [109,110]. Both are FDA-approved treatments for the treatment of RA. When IL-1ra was given intraperitoneally (i.p.) in a rat MCLT model 30 min before surgery and 3 h after surgery, IL-1ra significantly increased the percentage of granulated tissue in the healing region of the MCL as well as the number of M2 macrophages within and outside of the granulated tissue 5 and 11 days post-injury. IL-1ra also significantly decreased the protein expression of pro-inflammatory cytokines IL-12 and IL-1α at both of these time points in cells isolated from the injured MCL, and decreased IL-2 and IFN-γ, while it increased the anti-inflammatory IL-10 cytokine (at day 5 only). IL-1β was not inhibited by IL-1ra suggesting that IL-1 production continued despite treatment. IL-1ra significantly increased IL-6 at day 5 but at day 11 it decreased with IL-1ra treatment. IL-1ra had no effect on the mechanical behavior (ligament failure force, failure stress, and stiffness) of the healing ligaments vs. control (PBS)-treated injured rats [123], but the timing was likely too early to expect changes. In a similar study, this group showed that when IL-1ra was given subcutaneously (s.c.) in a rat MCLT injury model, both single and multiple s.c. daily injections (until day 4) of IL-1ra (600 ng) starting 18–24 h after injury decreased pro-inflammatory cytokines IL-1α, IL-1β as well as IL-6 in the MCL, but like the previous study, had no effect on mechanical properties compared to placebo-treated injured rats. However, multiple s.c. IL-1ra injections given daily did not provide additive effects and, in contrast, decreased the presence of the anti-inflammatory M2 macrophages, had no effect on the pro-inflammatory M1 macrophages and increased the pro-inflammatory IL-12 cytokine [126]. While these two studies [123,126] were focused on IL-1ra effects on injured MCL rather than injured cartilage, another study similarly showed that IL-1ra given s.c. (1 mg/day) for 4 weeks after closed articular fracture of the tibial plateau in C57BL/6 mice also resulted in significantly higher Mankin scores with frequent complete loss of articular cartilage and the presence of fibrocartilage as well as a lower tibial plateau bone volume fraction and higher levels of IL-6 in the fractured limb compared to saline-treated injured mice [110], indicating that multiple s.c. doses of IL-1ra given shortly after knee injury may promote deleterious effects. This study also showed that systemic long-term sTNFRII (i.p., 0.2 mg 3 times per week for 4 weeks) was similarly associated with the frequent appearance of fibrocartilage at the fracture site and loss of cartilage structure and proteoglycan staining and a greater loss of bone volume. A single i.a. injection of sTNFRII (0.3 mg) similarly led to significant degenerative changes as it significantly reduced the tibial plateau bone fraction vs. local saline treatment and, while a single i.a. injection of sTNFRII significantly decreased IL-6 in the synovial fluid in both the injured and uninjured knee joints, it significantly increased serum IL-6 and serum CTX-I, and increased the synovitis score. In contrast, in the same study, a single i.a. injection of IL-1ra (0.9 mg) significantly minimized the total cartilage Mankin score, reduced the synovitis score (i.e., the synovial lining thickness and cellular density), and, importantly, reduced serum and injured limb synovial fluid IL-6 concentrations 8 weeks after treatment [110]. Synovial fluid COMP levels (at 8 weeks post-injury) were not altered by any of the treatments. This suggests that IL-1ra is more beneficial than sTNFRII after knee trauma. Moreover, in a co-culture bovine articular superficial zone cartilage explant (1 mm thick)/synovium joint capsule (0.5 to 1 mm thick) model, continuous treatment with IL-1ra every 2 days for 24 days was required for inhibition of IL-1 induced GAG loss, NO production as well as collagen loss more than a single dose [127], indicating the short biological effectiveness of a single dose of IL-1ra.

Various studies have addressed the rapid clearance of IL-1ra and sTNFRII, and their short joint residence time using different i.a. release systems and have shown similar trends to those mentioned above when using IL-1ra vs. sTNFRII. One such study used cross-linked human elastin-like polypeptides (ELP) derived from human tropoelastin that form sponge-like micelle nanoparticles to encapsulate IL-1ra or sTNFRII, which was applied i.a. immediately after a closed articular tibial plateau fracture [109]. These uniformly sized ELP polypeptides have thermo-responsive properties, can be proteolytic degraded, and either entrap drugs or can be conjugated to drugs [128]. Using this approach, in vitro studies showed that both IL-1ra and sTNFRII could be released for up to 7.5 days in cell media and were detectable up to 5 days after i.a. injection in vivo but undetectable thereafter. ELP release of IL-1ra resulted in a moderate but not sustained effect in the ability of IL-1ra to decrease COMP in the synovial fluid and MMP-3 in the serum 4 weeks post-injury but was not capable of reducing Mankin or synovitis scores compared to untreated mice. ELP-mediated delivery of sTNFRII with or without IL-1ra appeared to increase cartilage Mankin and synovitis scores and decrease bone volume 4 and 8 weeks post-injury and the combination treatment also significantly elevated synovial fluid COMP levels at both time points compared to control-treated mice [109], again suggesting that sTNFRII treatment shortly after injury causes harmful effects. Another study used IL-1ra loaded superparamagnetic iron oxide PLGA microspheres, which release greater than 90% of IL-1ra within the first 3 days of loading, and showed that compared to i.a. injection of bolus IL-1ra, PLGA microsphere-mediated release of IL-1ra (given on days 7, 14, 21, and 28 following injury) prevented synovial inflammation, cartilage injury and loss of C-telopeptide fragments of collagen type II (CTX-II) in the urine, measured 4 weeks after ACLT in rats. In a human lymphocyte proliferation assay, the PLGA IL-1ra release system also dose-dependently inhibited IL-1β-induced lymphocyte proliferation, and in IL-1α stimulated bovine articular cartilage explants IL-1ra inhibited sGAG loss [124]. Another study showed that HA-chitosan (HA-CS) or CS-microspheres, which are capable of releasing IL-1ra for up to 15 days when treated with lysozyme, significantly improved the IL-1β-induced changes in cell viability and apoptosis, and inhibited GAG, prostaglandin E2 (PEG2) and ${\mathrm{NO}}_{2}^{-}$ production in rat chondrocytes. Interestingly, the release of IL-1ra from HA-CS microspheres was slower compared to IL-1ra released from CS-microspheres [125], indicating the importance of the material/structure of the delivery system in the kinetics of IL-1ra or another anti-inflammatory drug release. These findings show that i.a. inhibition of IL-1 pathways via IL-1ra may provide a therapeutic approach for reducing or preventing joint inflammation and degeneration following knee trauma [110,123,124,125] (as summarized in Figure 3), while TNF-α inhibition using sTNFRII may be detrimental [109,110].

Another approach uses surface-treated glass beads to physicochemically enrich IL-1ra from the patient’s blood to obtain autologous conditioned serum (ACS), which contains extremely high levels of IL-1-ra as well as growth factors including transforming growth factor-beta (TGF-β), platelet-derived growth factor (PDGF) and insulin-like growth factor 1 (IGF-1) as well as low concentrations of anti-inflammatory cytokines IL-4 and IL-10 [129,130]. Different formulations were developed (e.g., Orthokine^®^, Arthrokinex^®^, IRAP^®^) and several studies have shown that six consecutive i.a. injections, given over the course of 1 to 2 years, of ACS in symptomatic knee OA patients can improve pain and joint function (e.g., patient-reported knee mobility and function and global impression of change) for up to 2 years [131,132,133,134,135,136,137]. Some shortcomings of this approach include the short clearance time of cytokines [22,23,24,138] in vivo and the need for repeated i.a. injections over a short period of time. Moreover, factors such as the inflammatory status [139] as well as the incubation time with the surface-treated glass beads were shown to influence the ACS cytokine profile. Hence, some studies have shown that there is a small, but significant increase in pro-inflammatory cytokines IL-1β and TNF-α [130,140] at concentrations similar to those found in the synovial fluid after knee trauma or in early knee OA [3]. In the treatment of equine OA, i.a. ACS injections significantly increased IL-1ra and decreased the collagen degradative concentrations of C2C2 in the synovial fluid, while it had a moderate effect on decreasing synovial fluid cartilage matrix synthesis biomarkers (i.e., CPII, CS846) compared to baseline. However, ACS was given i.a. at two-day intervals for 42 days [138], which is unrealistic in the clinical setting. More pre-clinical studies are needed to confirm whether ACS is beneficial in delaying or preventing knee PTOA and whether it is capable of providing beneficial effects under those settings.

### 4.5. Anti-IL-6 Receptor Antibody

IL-6 is one of the most prominent and causal cytokines that is elevated at all stages of knee PTOA [3], making its inhibition an appealing potential target in the treatment or deterrence of PTOA. IL-6 is secreted by a large number of cells within the joint, including chondrocytes, osteoblasts, synovial fibroblasts, monocytes, and macrophages and is an important modulator of effector CD4 T cells functions (e.g., by inducing the development of IL-17 producing Th17 cells), which are all capable of contributing to a heightened immune response and chronic inflammation [141,142]. IL-6 exerts its effects on target cells via two mechanisms. The first mechanism, termed “classical” IL-6 signaling, is important in the acute phase of inflammation and occurs when IL-6 binds to both the membrane-anchored IL-6 receptor (mIL-6R), which lacks signal-transducing capabilities, and the (membrane-bound) glycoprotein 130 (gp130) receptor, which serves as the signal transducer of the mIL-6R/gp-130 complex. While the gp130 receptor is ubiquitously expressed on target cells, the mIL-6R is expressed primarily on inflammatory cells including neutrophils, monocytes, and macrophages as well as T cells. IL-6 also exerts effects through a second mechanism, termed “trans-signaling”, by interacting with the soluble IL-6 receptor (sIL-6R) that is released from cells expressing the mIL-6R through proteolytic processing in response to the increased presence of metalloprotease-disintegrins such as ADAM-17, also called tumor necrosis factor-α converting enzyme (TACE) due to its ability to shed TNF-α from the surface of cells, and ADAM-10, which increases in response to inflammatory cytokines such as IL-1β and TNFα and in OA [141,143]. The binding of IL-6 to sIL-6R has a two-fold effect: it increases the half-life of IL-6 and broadens the range of IL-6 responsiveness due to the universal expression of gp130 on all cell types. Therefore, while IL-6 binds to mIL-6R and sIL-6R with similar binding affinities, IL-6 “trans-signaling” is associated with the development of chronic inflammatory-associated diseases due to the ability of this receptor–ligand pair to interact with membrane-bound gp130 that is expressed by the majority of cell types in the knee joint [142,144].

Tocilizumab^®^ is a widely used humanized monoclonal antibody that binds to and competitively inhibits both the membrane-bound IL-6R and the sIL-6R [145]. Sarilumab^®^ is a more recently approved similar anti-IL-6 antibody with a much higher binding affinity [146]. While Tocilizumab is an accepted treatment for RA [147], to date, there is only one ongoing study on pain and function in patients with refractory hand OA (NCT02477059). There have not been any clinical studies using these IL-6 inhibitors to treat or prevent PTOA or knee OA, but with a large number of IL-6 pathway inhibitors available that target IL-6 via different mechanisms, this may change [142], especially since a few studies have shown that IL-6 inhibitors tested in in vitro chondrocyte and cartilage explant models as well as in animal models show protective effects [148,149,150,151,152,153]. Hence, systemic blockade of IL-6 using anti-IL-6 receptor antibodies or an inhibitor of the IL-6 signaling pathway (given once a week i.p. for 6 to 8 weeks beginning one day after a knee injury) reduced the severity and pathological phenotype in the medial meniscus and ACLT mouse injury models by preventing the production of IL-6 from subchondral bone mesenchymal stem/stromal cells (MSCs), decreasing cartilage lesions, subchondral bone sclerosis and synovitis [148,149]. Anti-IL-6 Fab fragments, which are smaller in size (48 kDa) than full-length antibodies and can penetrate articular cartilage unlike full-sized antibodies, also reduced the aggrecan degradation and partially prevented GAG loss in mechanically injured bovine and human cartilage explants exposed to a combination of IL-6, IL-6R and TNF-α [150,151]. An in vitro chondrocyte study showed that Tocilizumab decreased MMP-9 in the C28/I2 immortalized human chondrocyte cell line [152] and inhibited apoptosis induced by IL-6 in rat articular chondrocytes [153]. This suggests that IL-6 inhibition reduces synovial inflammation and cartilage lesions and could also inhibit apoptosis. However, more studies are needed to elucidate whether IL-6 inhibition is a viable option in delaying or preventing PTOA.

### 4.6. Anti-Inflammatory Cytokines IL-4, IL-10 and IL-13

IL-4, IL-10 and IL-13 are pleiotropic cytokines that function primarily by suppressing a pro-inflammatory environment through a negative regulatory autocrine and paracrine feedback loop. These anti-inflammatory cytokines are produced by several cell types including monocytes, macrophages and T helper 2 (Th2)-polarized T cells [154]. While no compensatory pathways exist for IL-10, IL-4 and IL-13, these three cytokines share some key features including binding to similar cell surface receptor subunits and activation of similar signaling pathways. However, despite some similarities and overlapping effects, they also elicit unique biological effects [154]. For instance, IL-10 gives rise to a population of regulatory macrophages that act as antigen-presenting cells for, e.g., local clearance of apoptotic cells, while IL-4 and IL-13 give rise to functionally and biochemically different wound healing macrophages [155,156], which could both regulate the response to a knee injury. IL-10 is also considered a gatekeeper of fibrotic and anti-fibrotic signaling [157]. As discussed in detail below, many studies suggest that these anti-inflammatory cytokines could be used to regulate inflammation in the knee joint as well as provide chondrocyte and cartilage tissue-protective effects.

IL-10 is significantly decreased in patients predisposed to developing severe knee OA after knee trauma [158]. Moreover, risk factors for OA such as obesity and diabetes mellitus are associated with decreased IL-10 levels [159]. Remarkably, MRL/MpJ mice, which have a superior tissue regenerative and healing response compared to other mouse strains (e.g., C57BL/6), do not develop PTOA following intra-articular fracture, which was attributed to the higher circulating IL-10 and IL-4 and lower IL-1β levels in these mice [160,161,162,163]. Interestingly, a single-nucleotide polymorphism (SNP) in the IL-4 receptor (IL-4R), which decreases the strength of IL-4 signaling, results in higher serum IL-17 levels and Th17 cells in RA patients vs. healthy individuals [164], suggesting that this specific IL-4R allele may allow unrestricted IL-17-mediated inflammation. Whereas there have not been any studies that have associated the IL-4R SNP with OA or PTOA, IL-17 was linked as a credible causal factor in inducing knee PTOA [3].

IL-4, IL-10 and IL-13 cytokines are absent in healthy synovial fluid and healthy cartilage tissue [165,166,167,168], but within 24 h of knee trauma IL-10 significantly increases in the synovial fluid and then concentrations taper off and return to baseline or below detectable levels two weeks after injury [166,167,168]. While IL-10 is locally present in only the first few days after knee trauma, both IL-4 and IL-13 are not detectable in the joint, suggesting that these protective cytokines are largely absent in the early stages after knee trauma and at a critical stage when regulation of inflammation may be needed to prevent progression towards PTOA. Receptors for these cytokines are highly present in cartilage tissue [169] and found on chondrocytes [170,171,172,173,174,175], synovial fibroblasts [176,177], synovial macrophages [178] as well as peripheral blood-derived monocytes, macrophages and T cells [178,179,180]. Optimal induction of IL-4, IL-10 or IL-13 anti-inflammatory cytokines requires integration of multiple signaling platforms that leads to their amplification. Although their transcriptional regulation has been intensely studied, the existence of multiple gene programs and autocrine/paracrine signaling pathways confound their regulation [181,182]. Therefore, the underlying spatiotemporal framework that dictates cell type- and patient-specific IL-4, IL-10 and IL-13 responses could be paramount to understanding their role in regulating and/or preventing chronic inflammation in the setting of post-traumatic knee joint inflammation and in harnessing their therapeutic potential. Although these anti-inflammatory cytokines are elevated in the synovial fluid and/or serum in the already progressed disease state (i.e., in clinically diagnosed OA and PTOA) [183,184,185,186,187,188,189], likely in an attempt to counteract chronic inflammation, it may be unlikely that their immunosuppressive and potential cartilage regenerating effects are sufficient to counterbalance late OA as OA chondrocytes exhibit a reduced response [172].

Interestingly, whereas there have not been any PTOA clinical studies using these anti-inflammatory cytokines, many preclinical models show that these cytokines not only modulate the inflammatory response but additionally stimulate chondro- and cartilage-protective effects. Accordingly, it was shown that continuous bolus IL-10 treatment, after injurious compression of mature bovine articular cartilage, can stabilize and promote a chondrogenic regenerative phenotype by increasing expression of chondrogenic markers COL2A1, ACAN, SOX9, decreasing the expression of COL1A1 and COL10A1, preserving the matrix and significantly reducing the injury-related cell death, release of GAG and NO and expression of MMP-3, -13, ADAMTS-4 and NOS2 [190,191]. Similarly, bolus IL-10 had beneficial effects on TNF-treated meniscus explants. Administration of IL-10 for 72 h significantly prevented the TNF-α-related cell death, release of NO and NOS2 expression, release of GAG and aggrecan neoepitope fragments and IL-10 reduced the expression of MMP-3 and -13 as well as ADAMTS4 and COL10A1 [192]. Bolus IL-10 also promoted chondrogenesis in a clinically applied collagen scaffold for ACI treatment containing human chondrocytes [190]. While ACI is usually performed to repair large-scale cartilage defects, in some rare cases it is also being used for the treatment of early OA. Therefore the chondrocytes used in [190] were most likely healthy chondrocytes. This suggests the possible use of IL-10 within other biomaterials to foster chondrogenesis for large-scale cartilage defect repair and for regenerating effects after knee trauma.

Others have shown that intravenous (i.v.) administration of a single low dose of recombinant human IL-10 was shown to be safe in healthy volunteers but it decreased blood lymphocytes (CD2, CD3, CD4, CD7, and CD8 positive cells) by 25% 3–6 h after the injection [23,193]. In ex vivo experiments, IL-10 also suppressed the production of bacterial LPS-induced TNF-α and IL-1β in whole blood. However, as i.v. treatment and high or repeated long-term doses could lead to unwanted side effects, localized approaches that concentrate anti-inflammatory cytokine therapies could tailor the effect exclusively within the knee joint. IL-10 was used in several clinical trials for various inflammatory diseases such as RA, Crohn’s disease, inflammatory bowel disease and psoriasis [194]. A current phase 1 clinical trial (NCT03477487) is studying the safety and efficacy of XT-150, a plasmid DNA encoding a variant of human IL-10 for treatment of severe OA to treat knee pain. A single i.a. injection is proposed with a six-month follow-up using KOOS and WOMAC scales to assess OA severity. As the study was only recently completed, results are not yet published.

In vitro studies on chondrocytes have shown that IL-10 can counterbalance the effects of TNF-α and IL-1 effects by protecting chondrocytes from cell death, maintaining cell proliferation, inhibiting IL-1β, nitric oxide synthase (NOS2) and MMP (e.g., MMP-3) expression [195,196,197], comparable to what was observed in TNF-α-treated bovine meniscus tissue [192]. All of the aforementioned studies demonstrate that IL-10 alone provides multi-factorial protective effects in healthy, as well as in OA chondrocytes and cartilage tissues (Figure 3). A recent study showed that while IL-10 was also capable of significantly decreasing COL1 expression in OA chondrocytes under hyperinsulinemia conditions, IL-10 was not as effective in enhancing proteoglycan synthesis or stimulating a regenerative phenotype under hyperglycemia or hyperinsulinemia conditions [198]. Even though inflammatory effects were not assessed in this study, this highlights the potential importance of investigating treatment strategies in co-morbidity studies.

IL-4 is also associated with chondrocyte protective effects. For example, IL-4 protected OA chondrocytes against IL-1β or LPS by decreasing the expression and production of CXC chemokines (CCL5/Rantes, macrophage inflammatory protein-1 alpha and beta (MIP1α, MIP1β) and ECM–degrading enzymes MMP-13 and ADAMTS4, prevented NO production and increased proliferation of chondrocytes [170,199,200,201,202]. IL-4 also reduced proteoglycan degradation and increased proteoglycan synthesis in IL-1α, TNF-α, the combination of IL-1α, TNF-α, LPS or IL-17 treated bovine cartilage explants [203,204]. In young and healthy rat chondrocytes, IL-4 protected the cells from excessive mechanical stress and resulting cartilage degradation exhibited by a dose-dependent increase in aggrecan and COL2 expression and a decrease in NO production [201,205]. Intra-articular administration of IL-4 in OA rats showed similar protective effects exhibited by decreased articular cartilage degradation and aggrecan loss and a significantly lower number of NO-positive chondrocytes [201].

Since IL-4 and IL-10 signal through different signaling pathways [181,182], it is thought that the combined administration of these two cytokines could exert different and potentially additive or synergistic effects [154]. Therefore, several studies have investigated the use of these cytokines alone or in combination under trauma-like conditions. Joint bleeding (hemarthrosis) routinely occurs after an acute knee injury in up to 98% of knee injuries [206,207,208,209,210] with volumes reaching up to 100% volume/volume *(v*/*v*). While blood entering the joint is typically cleared within a week if the bleeding stops [210], exposure of healthy articular cartilage tissue to 50% *v/v* of blood for a short period of time is capable of inducing inflammation as well as prolonged cartilage damage [169,211,212]. Several studies have assessed the effects of soluble IL-4 and/or IL-10 alone or application of an IL4-10 fusion protein against bleeding-associated trauma-like injury. IL-4 or IL-10 alone had dose-dependent effects by increasing proteoglycan synthesis and preventing its release, and by decreasing concentrations of IL-1β, TNF-α and IL-6 and chondrocyte apoptosis in cartilage explants obtained from healthy joints exposed to (50% *v*/*v*) whole blood or a combination of red blood cells and mononuclear cells (50% *v*/*v* equivalent to their amount in whole blood) [169,211,213], suggesting that IL-4 and IL-10 are both capable of modifying the effects of mononuclear cells. IL-4 and IL-10 also had comparable protective effects in degenerated joints with end-stage hemophilic arthropathy [212]. In a side-by-side comparison in a healthy human cartilage/blood injury using soluble IL-4 or IL-10 alone or together as a bolus or as an IL-4-10 fusion protein, all treatments similarly significantly diminished IL-1β and IL-6 levels to the control level. However, the combination of IL-4 and IL-10 (soluble or as a fusion protein) was more effective in increasing proteoglycan synthesis (by 2-fold) compared to IL-10 alone. The IL-4-10 fusion protein also reduced IL-1β and IL-6 production by CD14+ monocytes that had been stimulated with lysed red blood cells [211]. Another study in mice showed that co-injection of IL-1 with IL-4 and IL-10 into the knee joint of mice dose-dependently attenuated the IL-1–induced inhibition of proteoglycan synthesis. This study also showed that preincubation of mouse patellar cartilage explants with IL-4 followed by stimulation with either IL-1 or IL-17 resulted in increased proteoglycan synthesis and decreased NO levels but that IL-4–pretreated cartilage was less sensitive to IL-17–induced inhibition compared to IL-1 [204]. In healthy cartilage, the expression of IL-4(γc) and IL-10 receptors increased when the cartilage was exposed to blood and in the absence of blood, the beneficial effects on cartilage proteoglycan turnover did not take place [169]. Although they did not measure the effects of IL-13 in these studies, they did show that the IL-13 receptor was present and was not modified by the absence or presence of blood. This group also showed that in a hemophilia repeated joint bleed mouse model, that while a single bolus i.a. injection of IL-4 and IL-10 was not as effective in modifying cartilage degeneration or synovial inflammation in the knee joint after a single joint bleed [213], four consecutive i.a. IL-4-10 fusion protein injections ameliorated cartilage damage better than bolus injection of soluble recombinant murine IL-4 and IL-10 alone [211] and decreased pain in a canine groove in vivo model of knee PTOA [174,214]. However, neither treatment was capable of controlling synovial inflammation at day 35 after the first joint bleed in hemophilia mice [211]. These data show that under trauma-related conditions, IL-4 and IL-10 may control the post-injurious environment by preventing chondrocyte death and structural damage such as GAG loss, and by limiting inflammation in healthy individuals and that the combination of IL-4 and IL-10 may be more effective than each of these alone.

IL-13 is another cytokine that was shown to have anti-inflammatory and cartilage protective characteristics. An in vivo mouse study showed that local gene transfer of IL-13 was capable of reducing severe cartilage destruction by decreasing MMPs, IL-1 and preventing loss of aggrecan in an immune-complex-mediated arthritis model [215]. In another study, IL-13 inhibited the synthesis of IL-1β and TNF-α and MMP-3 and stimulated the production of IL-1ra in the OA synovial membrane treated with LPS and in synovial fibroblasts IL-13 decreased IL-1 binding, likely due to its ability to increase the production of IL-1ra [216]. Sustained release of IL-13 from microspheres was also capable of significantly inhibiting NO production in mouse OA chondrocytes challenged with IL-1β or LPS [199]. These data suggest that IL-13 may similarly have protective effects in PTOA. More studies are needed to demonstrate whether IL-13 plays a pivotal role in PTOA prevention.

Because the half-life of a bolus injection of IL-4, IL-10 and IL-13 is minutes to hours [22,23,24], these cytokines are rapidly cleared and require multiple injections. Besides the IL4-10 fusion protein approach discussed above, other approaches were developed to deliver anti-inflammatory cytokines in vivo, specifically two different gene therapy approaches [217,218] and a biomaterial-based sustained anti-inflammatory mediator release system approach [199]. In a PTOA model, i.a. injection of retrovirus-mediated genetically modified rabbit synoviocytes into rabbit knee joints containing human IL-10 and IL-1ra showed that gene delivery of both genes combined resulted in a stronger chondroprotective effect by blocking cartilage degradation and decreasing the loss of proteoglycans [217], while, in another study, IL-10 gene therapy of transduced human RA synovial cells or monocytic THP1 cells under a CXCL10 responsive promoter (CXCL10p-IL10 vector) reduced the production of inflammatory cytokines in response to stimulation with LPS [218]. In a 3D micro mass culture containing enzymatically digested human OA synovial tissue mixed in with Matrigel, in response to TNF-α or LPS stimulation, a single bolus of IL-10 was incapable of reducing IL-1β or TNF-α in contrast to treatment with the CXCL10p-IL10 vector, which significantly reduced the gene expression of both IL-6 and IL-1β but had no effect on the mRNA expression of TNF-α [219]. We recently demonstrated that bioresponsive gelatin microspheres, which have a net negative charge, could be used to sequester IL-4, IL-10 and IL-13 cationic cytokines and that IL-4 and IL-13 loaded microspheres co-cultured with mouse OA chondrocytes reduced NO production by up to 80%. Because of their material properties, they are responsive to proteolytic enzymes (i.e., MMPs) typically expressed during inflammation and preferentially degraded by inflammatory cells (e.g., M1 macrophages) and not by other non-inflammatory cells [220] and could allow on-demand and spatiotemporally controlled release of anti-inflammatory cytokines within the joint, thereby increasing the joint residence time and tissue-protective effects [199]. Overall, the data discussed in this section suggest that IL-10 possesses all four main therapeutic potentials against PTOA development due to its anti-inflammatory, anti-degenerative, anti-apoptotic and pro-regenerative effects and that IL-4 provides similar effects but the effect on apoptosis was not measured (Figure 3). Studies also suggest synergistic effects when using IL-4 in combination with IL-10. Synergistic combinations of anti-inflammatory drugs could help overcome toxicity and other side effects associated with high doses of single drugs and improve therapeutic selectivity [221] due to the differential regulation and expression of particular genes and unique effector functions [178]. IL-13 studies also point to anti-inflammatory and anti-degenerative effects but models of cartilage injury using IL-13 certainly require further study.

### 4.7. Complement Inhibitors

The complement system is a complex, multifaceted protein network and an essential component of the innate immune system and represents the first line of defense against any possible threat. It is activated by the classical, alternative and the lectin complement pathways, and not only protects against microbial infections and plays a regulatory role in eliminating cellular debris and stimulating adaptive immunity such as B and T lymphocytes, but it is also capable of inducing a state of perpetual inflammation that can lead to local and systemic tissue injury under adverse circumstances (e.g., in ischemic/hypoxic conditions) [222,223]. In the human arthritic joint, complement proteins are produced by chondrocytes, synoviocytes, macrophages as well as subchondral bone osteoblasts in response to the combination of blunt mechanical injury and exposure to human serum or pro-inflammatory cytokines IL-1β or TNF-α [45,224,225,226,227,228,229,230,231], the main cytokines involved in PTOA knee progression [3]. While each of the three complement pathways are activated by different conditions, all three pathways result in a proteolytic cascade that leads to i) formation of a pro-inflammatory environment, ii) the production of C3 and deposition of large amounts of C3b on target cells that acts as an opsonizing signal for phagocytic ingestion and subsequent killing, and iii) formation of the lytic membrane attack complex (MAC, sometimes referred to as terminal complement complex or TCC). Complement activation products of the classical/lectin (C4d), alternative (C3bBbP) and common pathway (soluble TCC) are significantly increased in the synovial fluid for up to 1.5 months following knee trauma and, while the levels of these complement components decrease over time, C4d remains elevated up to 10 years after a knee injury, demonstrating the unremitting activation of the complement system following trauma to the knee [207]. C3a and soluble TCC are also highly elevated in the synovial fluid of patients diagnosed with early knee OA (i.e., with symptoms of OA less than 1 year) [232,233] and inhibitors of the complement system are significantly decreased in both early- and end-stage OA [233]. In addition to the synovial fluid, in OA, complement activation products (C3/C3a, C4a, C4d, C3bBbP, factor B, and soluble TCC) are localized to the synovial membrane and the articular cartilage [207,232,233,234,235,236].

Considering that complement appears to play a role in PTOA progression in the knee [3,207,232,233,234,236,237], there are surprisingly few OA or PTOA studies that have clinically investigated local inhibition of the complement system. At present, there are two complement-inhibiting products that are clinically available. The first is a C1 inhibitor (C1INH) and the second, a monoclonal antibody against C5 (Eculizumab, sold under the name Soliris^®^) that inhibits cleavage of C5 into C5a and C5b and thereby prevents the formation of the MAC or TCC. The C1INH was tested in ischemia-reperfusion mediated injuries, while Eculizumab was tested in other inflammatory diseases and was proposed for treatment of rheumatic diseases such as RA or systemic lupus erythematosus (SLE) [237,238,239].

From the few studies that have used complement inhibitors, evidence suggests that they may potentially provide beneficial effects in PTOA [45,233,237,240,241]. Wang et al. was the first to investigate the importance of the complement system in OA progression and showed that 5 months after knee joint injury (medial meniscectomy), C5-deficient mice, which are incapable of forming the terminal MAC, were protected against degenerative effects and chondrocytes expressed significantly lower mRNA concentrations of leukocyte chemoattractants, MMPs (-3, 13, and 14) and ADAMTS-4 and -5, likely due to a lack of MAC-mediated signaling when compared to wildtype injured mice. Treatment (given i.v.) with CR2-fH, a fusion protein that inhibits activation of both C3 and C5, or a C5 neutralizing monoclonal antibody (given i.p.) was capable of significantly attenuating cartilage degeneration in the mouse knee joint after medial meniscus injury by 33% and 40%, respectively [233]. A C5-blocking antibody was also shown to upregulate COL2 in human OA cartilage explants suggesting that the chondrogenic phenotype could be potentially stabilized by blocking the accumulation of C5b-9 on cells [237]. TCC inhibitors also prevented TCC deposition and hemolytic activity on the surface of OA chondrocytes in a simulated injury model. Hence, treatment of mechanically injured OA cartilage tissue exposed to (30% *v*/*v*) human serum and cartilage homogenate with a C5b-9 inhibitor or a combined C9/alternative pathway inhibitor for 4 consecutive days after injury significantly decreased expression of IL-8, CXCL1, MMP-13 as well as the expression of several apoptotic and necroptotic markers [45]. In RA models, an anti-C5 monoclonal antibody fused to a peptide that allows homing to inflamed synovium by binding to the synovial microvascular endothelium in inflamed knee joints prevented synovial inflammation and localized production of IL-6 and TNF-α, as well as C9 deposition, but did not inhibit circulating levels of C5 [240]. This same group also showed that i.a. injection of a DNA vector encoding for an anti-C5 neutralizing antibody caused localized inhibition of C5 and reduced C9 deposition in the synovial tissue and synovial inflammation in the knee joint [241]. Therefore, although systemic complement inhibition was shown to be safe and feasible in treatment of other types of diseases [238], localized anti-complement therapies such as those discussed could be tailored to PTOA thereby concentrating the drug within the knee joint, while preventing systemic side effects.

### 4.8. Tranexamic Acid (TXA)

As discussed in this review, bleeding into the joint often occurs for up to 1 week after knee trauma [206,207,208,209,210] and causes inflammation-mediated cartilage damage [45,206,207,208,210,242,243]. Tranexamic Acid (TXA) is commonly used in orthopedic surgical settings to reduce excessive bleeding. Surprisingly, i.a. administration of TXA before and immediately after total joint replacement in patients with primary knee OA resulted in negative effects by significantly increasing concentrations of plasma TNF-α, monocyte chemoattractant protein-1 (MCP-1), which regulates migration and infiltration of monocytes and macrophages [167], and IL-4 two days after administration [244]. Long-term effects were not assessed in that study. While the previous study used TXA in patients with an already advanced disease state, a recently launched study (NCT03552705) uses TXA to determine its possible impact on knee PTOA preventative effects (i.a. bleeding and inflammation). This study combines a 5-day oral dose with i.v. administration of TXA during reconstructive ACL surgery within 4 days of unilateral ACL injury. The outcome measures of this study are synovial fluid IL-1 concentrations at day 5 as well as KOOS scores and quantitative MRI 6 months and 2 years after surgery. Although it was not included as a primary or secondary outcome measure, it would be interesting to determine if TXA counteracts blood-induced complement activation at early and late time outcome points and prevents early PTOA since complement activation products are present in the acute/subacute phases of injury as well as in early and late knee PTOA [3] and the combination of trauma and blood exposure increases the accumulation of complement activation and deposition on chondrocytes early after injury and induces pathological effects [45,207,245].

## 5. Possible Sustained Delivery Approaches to Prevent or Delay Knee PTOA

As summarized in Table 2, there are a few studies that have addressed the rapid clearance of corticosteroids (dexamethasone and TA) and the immunoregulators (IL-1ra, sTNFRII, IL-4, IL-10 and IL-13) and their short joint residence time using various sustained delivery approaches. The development of these different types of anti-inflammatory sustained delivery systems is encouraging and may improve the beneficial effects, especially since the data suggest that sustained delivery approaches are capable of regulating inflammation and cartilage degeneration over a longer time period and, thus, are more beneficial, compared to the respective single or multiple consecutive treatments. This suggests that these therapeutic approaches may enable better or prolonged control of early PTOA pathology. A single administration of sustained delivery drug systems can overcome the rapid clearance from the synovial space [19,20], avoid repeated injections that damages articular cartilage [68], and mutually allow targeted therapeutic delivery. Still, only a few studies have addressed early disease prevention using such approaches. Therefore, more extensive analysis is still needed for sustained delivery systems to fully exploit their potential for preventing knee PTOA, including crucial aspects such as the delivery method, the dosage, the timing of application, the length of drug delivery needed, and whether they should penetrate into the cartilage or remain on at the interface between the synovial fluid and cartilage tissue. Such points should be tested in various PTOA knee joint animals and combined injury/inflammatory articular cartilage explant models that accurately simulate acute knee injury and inflammation such as those described in [3] to unravel their “true” therapeutic efficacy. Additionally, the effects on the different cell types within the knee joint and cross-talk between those cell types under simulated models that incorporate synovial fluid or synovial fluid-like conditions [246] as well compressive conditions that mimic physiological joint loading or rehabilitation regimens would ideally help dissect how anti-inflammatory therapeutic approaches could be used for inducing repair and regulating inflammation under such conditions.

## 6. Discussion

Since PTOA occurs in younger patients compared to other forms of OA [6,15,247,248], it is evident that early strategies to prevent knee PTOA are needed well before clinical PTOA onset and subsequent disability. This review highlights the different types of anti-inflammatory therapeutic interventions that could be used to prevent or treat early disease progression towards PTOA of the knee joint, and that are capable of regulating multiple aspects of the disease, including inflammation and concomitant cartilage degeneration, with some treatment strategies, specifically HA and IL-10, also capable of inducing regenerative effects (Figure 3). Therefore, anti-inflammatory agents could be viable candidates to prevent PTOA. However, the question remains, what is the best therapeutic candidate to prevent PTOA and what is the best-sustained delivery approach? To date, there are no such studies that can answer this question and we would need head-to-head basic science studies that compare multiple therapeutic targeted interventions under simulated injurious/inflammatory conditions that reflect the post-traumatic knee joint, with particular emphasis on studies involving healthy cells or tissues since this would target the population that would benefit from such a treatment. Moreover, as shown in Table 1, current clinical trials still largely focus on pain management and the treatment of clinical knee OA, rather than PTOA prevention. Long-term cross-sectional longitudinal profile studies that include clinical, as well as basic science measurements measured from baseline injury and in a large population of patients with the same types of traumas could facilitate identifying the additional clinical benefits of disease-preventing PTOA drugs. Additionally, the inclusion of novel imaging technologies that are capable of detecting early pathology before structural and degenerative changes become irreversible, such as a non-destructive quantitative optical biopsy that uses a clinically available endomicroscope and combines an artificial intelligence (AI)-based random forest-based approach to diagnose and differentiates between healthy vs. early OA [249], could also help to determine the therapeutic effectiveness of current as well as future therapeutic interventions aimed at preventing or delaying PTOA.

Figure 2 provides an overview of the major cell types and the basic mechanisms and factors of the knee joint currently thought to contribute to the chronic feedback loop of inflammation and tissue degeneration. More recently, however, the emergence of single-cell RNA sequencing (scRNA-seq) has led us to redefine joint tissue-specific cell subpopulations within each of these tissues. For example, it has only recently emerged that there is large heterogeneity in synovial intima and subintima synovial fibroblasts and macrophages as well as the articular cartilage chondrocytes that form a broad spectrum of phenotypes depending on their anatomic distribution but also, importantly, on the disease state (i.e., in healthy vs. OA or PTOA) [26,27,28,29,30]. The integration of scRNA-seq and spatial transcriptomics opens up new opportunities to define the organization of cellular niches and crosstalk between these cell and tissue types that modulate cellular function [250]. The identification of these cell types opens up a whole new area of research to understand how these cells relate to PTOA progression and to uncover the cellular targets of new and existing therapeutic strategies in resolution of inflammation and tissue repair. Full characterization of the cell types and states affected by therapeutic interventions that are capable of regulating multiple aspects of disease progression may improve our understanding of, and ability to better prevent or treat PTOA.

In conclusion, this review elucidated multiple targets to counteract post-traumatic inflammation based on high-quality basic science and clinical studies. Surprisingly, the immune-modulatory and anti-degenerative effects of many of those targets have not yet been evaluated in a clinical trial and, thus, promising targets remain on the horizon.

## Figures and Tables

**Table 1:** The few clinical trials aimed at preventing or delaying knee PTOA.

Clinical Anti-Inflammatory Approaches to Prevent or Delay Knee PTOA					
Intervention	Trial Name	Patient Criteria	Study Design	Outcome	Benefit Observed
**Triamcinolone acetonide from PLGA microspheres (FX006, Zilretta^®^)**	Success of Long-acting Anti-inflammatories After Anterior Cruciate Ligament and Meniscal Injury (SLAM) (NCT04331002)	Elevated synovial fluid IL-6 remaining 4 weeks after ACL reconstruction with meniscal involvement (Age 18–40 years)	Single i.a. injection 8 weeks after ACL reconstruction in a Phase 2, randomized, quadruple blinded, parallel assignment, placebo-controlled study	Bone Shape, IKDC, KOOS Global ICOAP at baseline, 4 months, 1 year and 2 years after intervention; CTX-II levels only at baseline and 4 months	Recruiting
**Triamcinolone acetonide alone (Kenalog^®^-40) vs. Triamcinolone acetonide from PLGA microspheres (FX006, Zilretta^®^)**	Proof of Concept Study Comparing FX006 to Kenalog^®^-40 in Patients With Post-Traumatic Osteoarthritis of the Knee (NCT02468583)	Kellgren-Lawrence (KL) Grade 2 or 3 PTOA (Age 20–50 years)	Single i.a. injection in a Phase 2, randomized, quadruple blinded, parallel assignment study	Pain intensity score using NRS, WOMAC—(A1, B, C and Total), KOOS, PGIC, CGIC, % of responders according to OMERACT-OARSI criteria, time to onset of pain relief and average weekly and total consumption of rescue medication over 12 weeks after intervention	Data not yet available
**Recombinant human IL-1ra (Anakinra^®^)**	Study to Prevent Cartilage Damage Following Acute Knee Injury (NCT00332254) [25]	Onset of a sports-related ACL tear requiring surgery (Age 18–30 years)	Single i.a. injection within 4 weeks (a mean 15 ± 7 days) of knee injury in a Phase 1/2, randomized, quadruple blinded, parallel assignment, placebo-controlled study involving 11 patients	KOOS at baseline before treatment, 4 and 14 days after intervention; SF IL-1α, IL-1β and IL-1ra levels and serum HA at baseline and a mean of 35 days after treatment	Improvement in KOOS, decrease in SF IL-1α within 2 weeks of treatment
**Recombinant human IL-1ra (Anakinra^®^)**	Study to Early PTOA Following Acute Knee Injury (NCT02930122)	ACL tear and painful effusions (Age 14–40 years)	Single i.a. injection within 4 weeks of injury in a Phase 2 prospective, single-center, randomized, triple-blinded, placebo-controlled study	CTX-II levels from injury to time of surgery, KOOS scores and quantitative T1rho MRI 1 year after injury	Data not yet available

Abbreviations: ACL (anterior cruciate ligament), CGIC (Clinician Global Impression of Change), CTX-II (C-telopeptide fragments of type II collagen), HA (hyaluronic acid), ICOAP (Intermittent and Constant Osteoarthritis Pain Score), IKDC (International Knee Documentation Committee Subjective Knee Form), IL-1ra (interleukin-1 receptor antagonist), IL-1α (interleukin-1 alpha), IL-1β (interleukin-1 beta), KOOS (Knee Injury and Osteoarthritis Outcome Score), MRI (magnetic resonance imaging), NRS (Numeric Rating Scale), OMERACT-OARSI (Outcome Measures in Rheumatology and Osteoarthritis Research Society International initiative), PGIC (Patient Global Impression of Change), PGLA (poly(lactic-co-glycolic acid), SF (synovial fluid), WOMAC (Western Ontario and McMaster Universities Osteoarthritis Index) —A1 (pain on walking), —B (stiffness), —C (function).

**Table 2:** Possible sustained delivery approaches in preventing or delaying knee PTOA. In order to extend the release and the sustainability of different therapeutic agents used to treat or delay PTOA progression, different delivery systems of synthetic or natural biomaterials were tested, whereas only PLGA microspheres Zilretta^®^ is approved by the FDA to treat OA-related knee pain. Abbreviations: ACL (anterior cruciate ligament), ACLT (anterior cruciate ligament transection), IL-4 (interleukin-4), IL-10 (interleukin-10), IL-13 (interleukin-13), IL-1ra (interleukin-1 receptor antagonist), IL-1α (interleukin-1 alpha), IL-1β (interleukin-1 beta), MCL+MM (medial collateral ligament + medial meniscectomy), MCLT (medial collateral ligament transection), nuclear factor-kappa B (NF-kB), NO (nitric oxide), PG (proteoglycan), PGPS (peptidoglycan-polysaccharide (PGPS), PGLA (poly(lactic-co-glycolic acid), sTNFRII (soluble receptor for tumor necrosis factor type II).

Therapeutic Agent	Drug Delivery	Pre-Clinical and Clinical Studies	Effect	Company/ References
**Dexamethasone**	Lipid-based microspheres (TLC599, BioSeizer^®^)	Phase 2 (OA-associated knee pain)	Reduced pain	TLC, [67]
**Dexamethasone**	Avidin	Bovine cartilage explant (IL-1α) model PTOA (ACLT) rabbit model	Rescued IL-1α induced cell death and decreased cartilage degeneration Decreased inflammation and cartilage degeneration	[53,65,66]
**Triamcinolone Acetonide**	PLGA microspheres (FX006, Zilretta^®^)	FDA approved (OA-associated knee pain); Phase 2 (PTOA, ACL injury) Acute synovitis (PGPS) rat model	Reduced pain Results not yet published Decreased inflammation and cartilage degeneration	Flexion Therapeutics [70] NCT04331002 NCT02468583 [79]
**Triamcinolone Acetonide**	PEA microspheres	OA (type II collagenase) rat model; Acute synovitis (bacterial PGPS) rat model	Decreased inflammation but was not capable of decreasing cartilage degeneration	[77,78]
**IL-1ra** **with or without** **sTNFRII**	Elastin-like polypeptide (Anakinra^®^ vs. Etanercept^®^)	PTOA (MCLT) mouse model	IL-1ra decreased inflammation and cartilage degeneration sTNFRII had adverse effects on cartilage and bone and caused synovial inflammation	[109]
**IL-1ra**	PLGA microspheres (Anakinra^®^)	PTOA (ACL tear) rat model	Decreased inflammation and cartilage degeneration	[124]
**IL-1ra**	Gene therapy Adenovirus vector under with NF-kB-responsive promoter (FX201)	Phase 1 (Knee OA)	Results not yet published	NCT04119687
**IL-1ra**	HA-chitosan microspheres	Rat chondrocyte (IL-1β) model	Decreased inflammation and chondrocyte apoptosis	[125]
**IL-1ra and IL-10**	Gene therapy retrovirus vector	PTOA (MCL+MM) rabbit model	Decreased cartilage degeneration	[217]
**IL-4 and IL-10**	Fusion protein	Human OA knee cartilage and synovium; PTOA (groove) canine model	Decreased inflammation, improved PG turnover and reduced pain	[174]
**IL-4 and IL-10**	Fusion protein	Human healthy cartilage (50% *v/v* blood-injury model); PTOA (joint bleeding) hemophilic mouse model	Decreased cartilage degeneration	[212,213]
**IL-10**	Gene therapy lentivirus vector with CXCL10-responsive promoter	RA synovial cell and THP-1 monocyte cell line (LPS) models; Human OA synovial membrane/Matrigel 3D culture (TNF-α or LPS) model	Decreased inflammation	[218,219]
**IL-4, IL-13 or IL-10**	Gelatin microspheres	ATDC-5 mouse chondrocyte (IL-1β or LPS) model	IL-4 and IL-13 decreased NO production	[199]

## Data Availability

Not applicable.

## Abbreviations

ACAN	Aggrecan
ACLT	Anterior cruciate ligament transection
ACS	autologous conditioned serum
ADAMTS	A disintegrin and metalloproteinase with thrombospondin motifs
bFGF	Basic fibroblast growth factor
C1INH	C1 inhibitor
COLI	Collagen I
COLII	Collagen II
COMP	Cartilage oligomeric matrix protein
CTX-I	C-terminal crosslinked telopeptide type I collagen
CTX-II	C-terminal crosslinked telopeptide type II collagen
CXCL	Chemokine ligand
ECM	Extracellular matrix
ELP	Elastine-like polypeptide
GAG	Glycosaminoglycan
GlcNAc	N-acetylglucosamine
gp130	Glycoprotein 130
HA	Hyaluronic acid (HA)
IFN-ɣ	Interferon-gamma
IGF-1	Insulin-like growth factor 1
IL	Interleukin
IL-1ra	IL-1 receptor antagonist
IL-6R	IL-6 receptor
i.a.	Intra-articular
i.p.	Intraperitoneal
i.v.	Intravenous
iNOS	Inducible NO synthase
IGF-1	Insulin-like growth factor 1
kDa	Kilodalton
KOOS	Knee Injury and Osteoarthritis Outcome Score
LPS	Lipopolysaccharide
MAC	Membrane attack complex
MCLT	medial collateral ligament transection
MSC	Mesenchymal stem/stromal cells
MCP-1	Monocyte chemoattractant protein-1, also known as CCL2
MIP-1	Macrophage inflammatory protein-1
MMPs	Matrix metalloproteinases
*M*_W_	molecular weight
MRI	Magnetic resonance imaging
NO	Nitric oxide
NOS2	Nitric Oxide Synthase 2
NSAIDS	nonsteroidal anti-inflammatory drugs
OA	Osteoarthritis
PCM	Pericellular matrix
PDGF	Platelet-derived growth factor
PEA	Polyester amide
PGPS	peptidoglycan-polysaccharide streptococcal
PEG	Polyethylene glycol
PGE2	Prostaglandin E₂
PLGA	Poly (lactic-co-glycolic acid)
PTOA	Post-traumatic osteoarthritis
RA	Rheumatoid arthritis
s.c.	subcutaneous
sIL-6R	Soluble IL-6 receptor
sTNFRII	Soluble TNF receptor II
TCA	Triamcinolone acetonide
TCC	Terminal complement complex
TACE	Tumor necrosis factor-α converting enzyme
TGF-β	transforming growth factor-beta
TIMP	Tissue inhibitor of metalloproteinases
TNF-α	Tumor necrosis factor-alpha
Th	T helper
TSG-6	TNF-stimulated gene 6 protein
TXA	Tranexamic Acid
WOMAC	The Western Ontario and McMaster Universities Osteoarthritis
